# One-Pot Synthesis of Organically Intercalated Hectorite and Its Adsorption of Phenol from Wastewater

**DOI:** 10.3390/ma19153318

**Published:** 2026-08-04

**Authors:** Yunhan Zhao, Xueting Wang, Jinyang Chen

**Affiliations:** School of Environmental and Chemical Engineering, Shanghai University, 99 Shangda Road, Shanghai 200444, China; mrsteven777@163.com (Y.Z.); wang-xueting@shu.edu.cn (X.W.)

**Keywords:** hectorite, one-pot synthesis, octadecyl trimethylammonium chloride, phenol, adsorption

## Abstract

In this study, hectorite intercalated with octadecyl trimethylammonium ions was synthesized via one-pot synthesis, and the octadecyl trimethylammonium-modified hectorite was used as an adsorbent to remove phenol from an aqueous solution. The pH and content of the adsorbent were studied to determine the optimized conditions for the adsorption of phenol. The phenol removal rate attained for 50 mL of 100 mg/L initial phenol solution at pH 12 was about 92.3% when 0.5 g of adsorbent was used. As for the adsorption isotherm, the Langmuir and Freundlich models were appropriate. The adsorption kinetics were in accordance with the pseudo-second-order model, and the activation energy (Ea) was about 11.15 kJ/mol. The modified hectorite could be recycled and reused, maintaining a high adsorption amount after five cycles.

## 1. Introduction

Phenol is a common organic pollutant in water, and it is classified as a priority pollutant; it affects the taste and smell of water in concentrations as low as 0.5 mg/L, as pointed out by the US Environmental Protection Agency [1,2]. Thus, the removal of phenol from wastewater is very important, and there are many methods for this process, such as chemical oxidation [3,4,5], solvent extraction [6,7], membrane separation [8,9], biological treatment [10] and adsorption. Adsorption is a crucial method owing to its low cost and simple operation process [11,12,13,14,15,16,17,18,19,20].

Layered clay minerals such as montmorillonite have a large number of exchangeable ions and present excellent adsorption properties [21]. Direct adsorption with inorganic clay minerals is not feasible for organic phenol due to its hydrophilic nature. As for the adsorption of organic phenol from aqueous solutions, the interlayer inorganic cation should be replaced with an organic cation such as organic quaternary ammonium to increase lipophilicity [22,23]. In a previous study, common natural montmorillonite was modified via intercalation of hexadecyl trimethylammonium to adsorb phenol and benzene, and the maximum adsorption amounts were 4.44 and 35.8 mg/g [24]. The low adsorption amounts can be attributed to the modification mechanism involving the substitution of interlayer cations, where the original intrinsic cations cannot be completely replaced during the modification process.

In recent years, it has been found that hectorite (a type of montmorillonite with a nominal layer charge of 0.35 eq per formula unit (f.u.) caused by substitution of Li^+^ with Mg^2+^ in the octahedral sheet) has great cation exchange capacity [25], and its significant ion exchange ability no doubt results in equally high adsorption capacity. Therefore, the use of organically modified hectorite to adsorb phenol from aqueous solutions represents a promising research direction. A common method of hectorite modification utilizes the mineral’s adsorption and ion exchange properties. In this process, hectorite is first synthesized using a hydrothermal method, and subsequently, the organic components enter the interlayer to replace its existing cations. Another common synthesis approach is to mix the organic precursor solution with the suspension of hectorite directly, although this process is complicated and lengthy [26,27,28]. Furthermore, it is difficult for the organic components to enter the interlayer of hectorite, which limits the adsorption activity because the quantity of the organic components entering the interlayer is relatively low.

Therefore, we studied the synthesis of the organic intercalation modification of hectorite using the steam-assisted one-pot method. The organic intercalation hectorite was directly obtained after thorough mixing of the reactants of different mole ratios of lithium, magnesium, silicon and organically modified quaternary ammonium salt. Because the organic intercalation and the formation of hectorite occur simultaneously, the organic cations can easily enter the interlayer of hectorite. The modification of hectorite with quaternary ammonium cations increases its lipophilicity and layer spacing. This method not only eliminates the cleaning process for synthetic hectorite gel but also greatly reduces its drying cost. The phenol in aqueous solution was removed through adsorption using organically modified hectorite, and the isotherms and kinetics were studied.

## 2. Experiment

### 2.1. Materials

Lithium fluoride (LiF), active magnesium oxide (MgO), precipitated silica (SiO_2_), Octadecyltrimethylammonium chloride and phenol were all obtained from Sinopharm Chemical Reagent Co., Ltd. (Shanghai, China). Synthetic hectorite (Na_0.6_[Mg_5.4_Li_0.6_Si_8_O_20_(OH·F)_4_]) was obtained from Jufeng New Material Technology Co. (Anhui, China). Distilled water was also used in the study.

### 2.2. Synthesis of OTMA^+^/Hectorite

**Figure 1:**
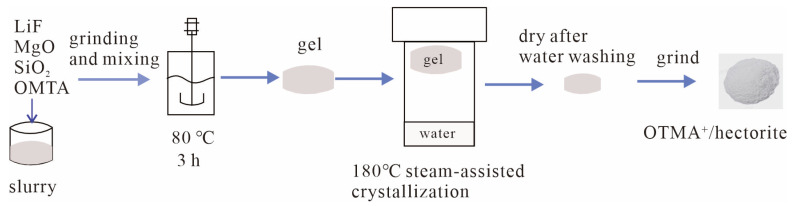
A schematic diagram of the synthesis process.

The synthesis process makes reference to previous methods and is shown in Figure 1 [4]. Firstly, a certain amount of LiF, active magnesium oxide (MgO), precipitated silica (SiO_2_) and octadecyl trimethylammonium chloride was ground and mixed to form a uniform slurry, and the mole ratio of Li:Mg:Si:OTMA was 1.2:5.4:8:0.6. Then, the slurry was thoroughly stirred at 1200 rpm and 80 °C for 3 h to obtain a gel. The gel was placed inside the top sieve of a Teflon-lined autoclave containing distilled water at the bottom for steam-assisted crystallization. The autoclave was closed and placed in a Muffle furnace at 180 °C for 36 h. After the crystallization period, the autoclave was taken out and quenched to room temperature. The product was collected, dried at 120 °C for 6 h and then ground, and finally sifted through a 300-mesh filter to obtain OTMA^+^/hectorite composite adsorbent.

### 2.3. Characterization

The XRD measurements were carried out using a multifunctional X-ray diffractometer with a Cu anode (Riken Electric Co., Ltd., Tokyo, Japan) running at 40 kV and 250 mA, scanning from 5 to 80° at 6°/min. FTIR spectra were obtained using the KBr pellet technique with an AVATAR 370 FTIR spectrophotometer (Thermo Nicolet Corporation, Waltham, MA, USA). Transmission electron microscopy (TEM) and scanning electron microscopy (SEM) images were obtained using a JSM-6700 Cold FE SEM device (Jeol, Tokyo, Japan) operating at an acceleration voltage of 20 kV after the samples were sputtered with gold. The BET surface area, pore volume and pore size were measured via N_2_ adsorption using an ASAP 2460 analyzer (Micromeritics Inc., Norcross, GA, USA). N_2_ adsorption was carried out at 77 K, and the samples were degassed at 523 K.

### 2.4. Adsorption of Phenol

Adsorption of phenol from aqueous solution was carried out in a thermostatic orbital shaker. The effects of variables, including pH, adsorbent dosage, initial phenol concentration, time and temperature, on the removal rate of phenol were studied. The pH of the solution was adjusted with 0.1 M HCl and 0.1 M NaOH.

After adsorption equilibrium, the phenol solution was centrifuged, and the concentration was measured using light of 510 nm wavelength with an ultraviolet–visible spectrophotometer. The adsorption amount of phenol was calculated according to Equation (1):

(1) $$q_{e}=\;\frac{(C_{0}-C_{e})V}{m}$$

where C_0_ and C_e_ are the initial and adsorption equilibrium concentrations of phenol in solution (mg/L), respectively. V is the volume of phenol solution (L), and m is the weight of OTMA^+^/hectorite (g).

The adsorption removal rate of phenol was calculated according to Equation (2).

(2) $$R=\frac{C_{0}-C_{e}}{C_{0}}\times 100$$

where C_0_ and C_e_ are the same as in Equation (1).

### 2.5. Recycling

A thermal desorption method was studied to recycle OTMA^+^/hectorite by heating to 190 °C under vacuum for 3 h to allow the phenol to be volatilized into the gas collection system. Then, the desorption OTMA^+^/hectorite was used again to adsorb the phenol in aqueous solution.

## 3. Results and Discussion

### 3.1. One-Pot Synthesis Reaction Mechanism

The common synthesis method of hectorite is hydrothermal reaction, and the typical equation is as follows.

LiF + MgCl_2_ + Na_2_CO_3_ + Na_2_O·nSiO_2_ → M_x_[Li_x_Mg_6-x_Si_8_O_20_(OH·F)_4_] (M = Na, Li)] (hectorite) + NaCl + CO_2_

Washing hectorite is very difficult because it is in a gel state in water. Furthermore, the cost of treating saline and alkaline wastewater is very high. The steam-assisted synthesis of this study is therefore critical, and its reaction equation is as follows:

LiF + MgO + SiO_2_ + H_2_O→ Li_x_[Li_x_Mg_6-x_Si_8_O_20_(OH·F)_4_] (hectorite)

Based on this reaction, raw materials of MgCl_2_, Na_2_CO_3_ and Na_2_O·nSiO_2_ are replaced by MgO and SiO_2_, and there is no other byproduct.

In the one-pot synthesis of hectorite with the replacement of interlayer cations by OTMA^+^, raw materials are mixed with octadecyl trimethylammonium chloride, and the reaction equation is as follows:

OTMA^+^Cl + LiF + MgO + SiO_2_ + H_2_O → OTMA^+^_x_[Li_x_Mg_6-x_Si_8_O_20_(OH·F)_4_] (OTMA^+^/hectorite) + LiCl

**Figure 2:**
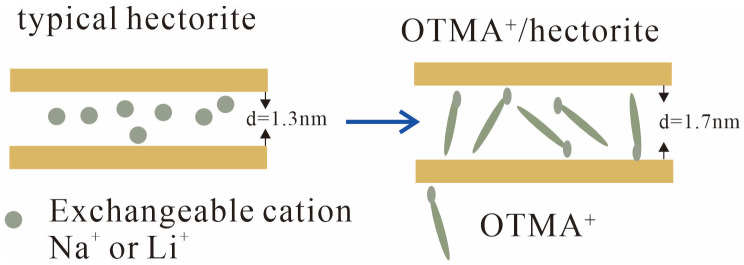
The schematic diagram of the exchange of interlayer cation in hectorite.

The one byproduct of LiCl is exchanged into high-pressure liquid water, and the gel is OTMA^+^_x_[Li_x_Mg_6-x_Si_8_O_20_(OH·F)_4_]. Because the long-chain organic cations replace the inorganic sodium or lithium cations, the interlayer spacing of hectorite increases from the traditional 1.3 to 1.7 nm. Figure 2 shows a schematic diagram of the increase in interlayer spacing after the substitution. As for this synthesis process, lithium acts as the interlayer countercation to form Li_x_[Li_x_Mg_6-x_Si_8_O_20_(OH·F)_4_], and interlayer lithium ions are substituted to obtain OTMA^+^_x_[Li_x_Mg_6-x_Si_8_O_20_(OH·F)_4_]. This procedure leads to a byproduct of LiCl. The process can also use the same molar amounts of NaF and LiF as starting reactants. Therefore, the corresponding products are Na_x_[Li_x_Mg_6-x_Si_8_O_20_(OH·F)_4_], OTMA^+^_x_[Li_x_Mg_6-x_Si_8_O_20_(OH·F)_4_] and NaCl. The dosage of lithium can thus be reduced.

The substitution of interlayer long-chain organic ions, which enlarges the interlayer spacing, not only increases the void ratio but also transforms the interlayer composition from inorganic ions to organic ions. This enhances the affinity for organic molecules and thereby greatly improves amounts of absorbed organic pollutants. This is the foundation for hectorite’s application as an adsorbent material for organic pollutants.

### 3.2. Characterization

**Figure 3:**
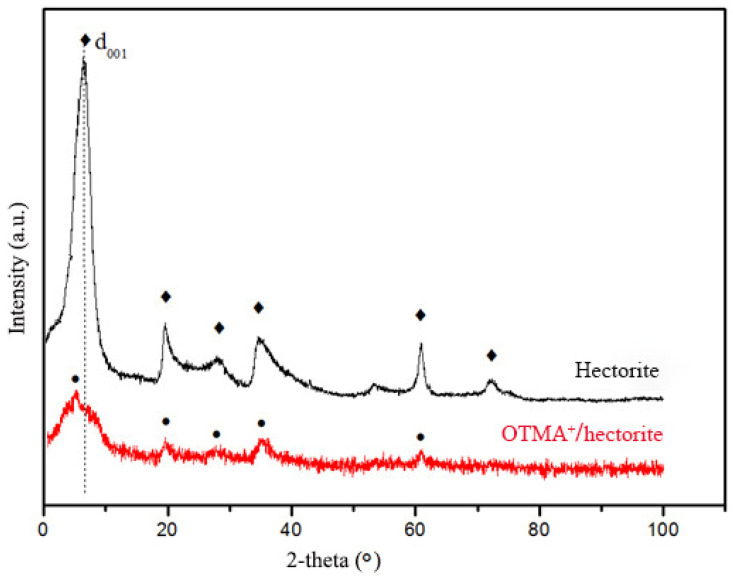
XRD powder patterns of hectorite and OTMA^+^/hectorite.

The XRD patterns of typical hectorite and OTMA^+^/hectorite are given in Figure 3. The hectorite shows six diffraction peaks at 6.72, 19.76, 29.01, 35.16, 53.28 and 60.8°, which correspond to planes (001), (110), (004), (200), (211) and (330) of typical hectorite. Modified OTMA^+^/hectorite has similar diffraction peaks, which indicates that OTMA^+^/hectorite retains the structure of hectorite. The diffraction peaks of OTMA/hectorite appear significantly less intense than those of the parent material, and the organically modified lithium laponite synthesized via steam exhibits reduced crystallinity after organic modification. Such loose structures should be more favorable for subsequent adsorption. With the entrance of OTMA^+^ into the interlayer of hectorite, the diffraction peak of plane (001) shifts from 6.72 to 5.38°, indicating that the basal spacing of 1.3 nm of hectorite is enlarged to 1.7 nm. The larger layer spacing after organic modification has a more obvious advantage for adsorption.

**Figure 4:**
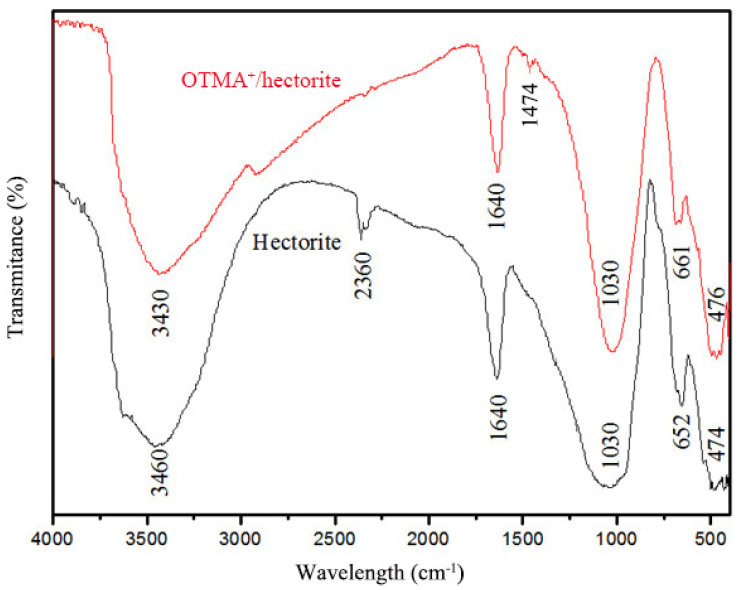
FT-IR spectra of hectorite and OTMA^+^/hectorite.

The FT-IR spectra of hectorite and OTMA^+^/hectorite are shown in Figure 4. The asymmetric stretching vibration of Si–O was found at 1030 cm^−1^ for hectorite and OTMA^+^/hectorite. The stretching band around at 3460 cm^–1^ is characteristic of the –OH group in hectorite [29], whereas the corresponding band of OTMA^+^/hectorite moves to 3430 cm^−1^, indicating that OTMA^+^ enters the interlayer of hectorite after modification. The bands around at 1640 cm^−1^ result from the angular vibration of adsorbed water [30]. The weak bands at 2922 cm^−1^ for OTMA^+^/hectorite are attributed to the C–H asymmetric stretching vibrations of OTMA^+^. The weak band of 1474 cm^−1^ is the bending vibration of –CH_3_ of OTMA^+^, and that of 2360 cm^−1^ should be the interlayer-adsorbed water. After organic substitution, the absorption peak corresponding to interlayer-adsorbed water weakens, indicating a reduction in the amount of adsorbed water. Mg–OH stretching vibrations should be observed at 652 for hectorite and 661 cm^−1^ for OTMA^+^/hectorite.

**Figure 5:**
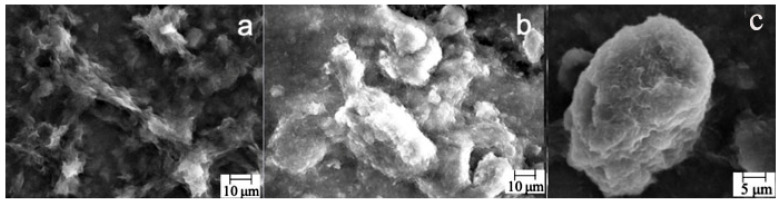
The SEM images of hectorite and OTMA^+^/hectorite: (**a**) hectorite; (**b**) and (**c**) OTMA^+^/hectorite.

The SEM images of hectorite and OTMA^+^/hectorite are shown in Figure 5. The surface properties of hectorite change from hydrophilic to hydrophobic due to the intercalation of the organic cations into the interlayer. When hectorite and OTMA^+^/hectorite were dispersed in ethanol, the former showed a layered structure, while the latter formed a platy and coral-like structure.

**Figure 6:**
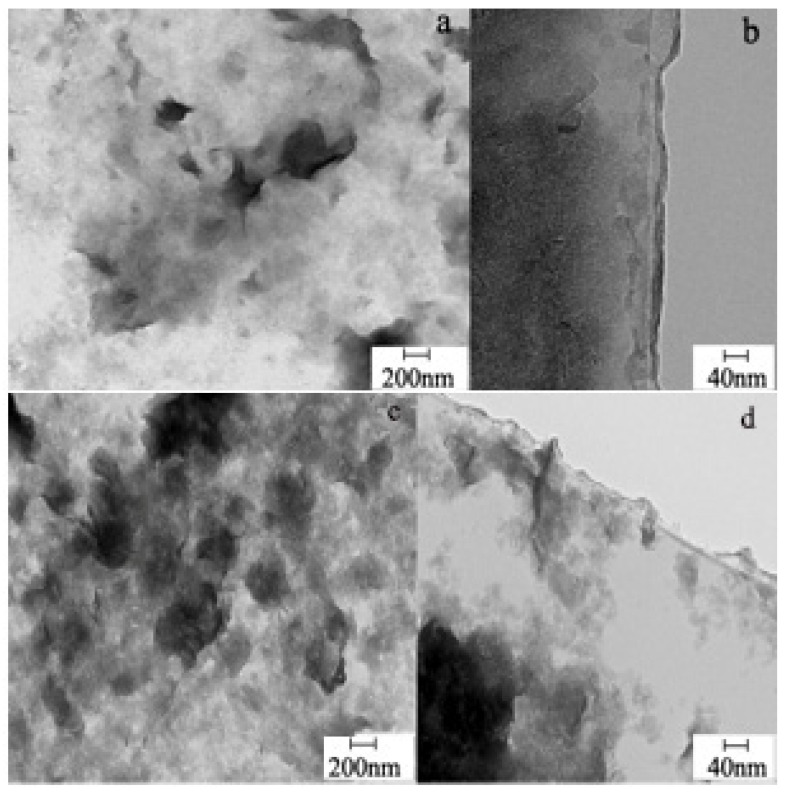
The SEM images of hectorite and OTMA^+^/hectorite: (**a**,**b**) hectorite; (**c**,**d**) OTMA^+^/hectorite.

The TEM images of hectorite and OTMA^+^/hectorite are given in Figure 6. The hectorite shows porous and lamellar structure, and the main structure is not changed after modification by OTMA^+^. There is more porosity with the entrance of OTMA^+^ into hectorite, and the increase in porosity provides more spaces for its adsorption.

**Figure 7:**
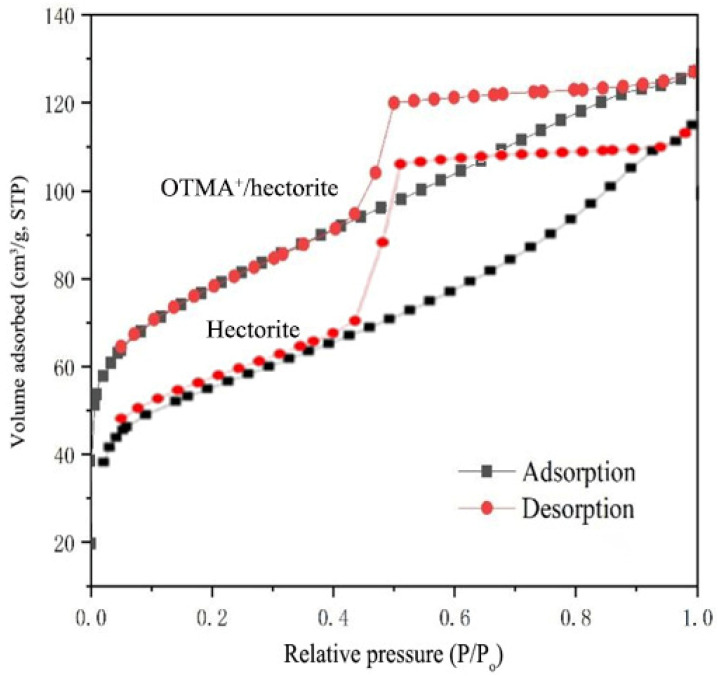
N_2_ adsorption/desorption isotherms of hectorite and OTMA^+^/hectorite.

The N_2_ adsorption–desorption isotherms of OTMA^+^/hectorite are shown in Figure 7. The isotherm plot is type IV, with H3 hysteresis loops attributed to the multilayer formation and capillary condensation in mesopores [31]. The BET surface area, pore diameter values and pore volume of OTMA^+^/hectorite and other similar aluminosilicate clay minerals are given in Table 1. They indicate that the pore diameter of OTMA^+^/hectorite has increased compared with hectorite. The S_BET_ of OTMA^+^/hectorite is significantly larger than that of montmorillonite modified by polyacrylamide, and the OTMA^+^/hectorite should have excellent adsorption properties.

### 3.3. Adsorption Condition

**Figure 8:**
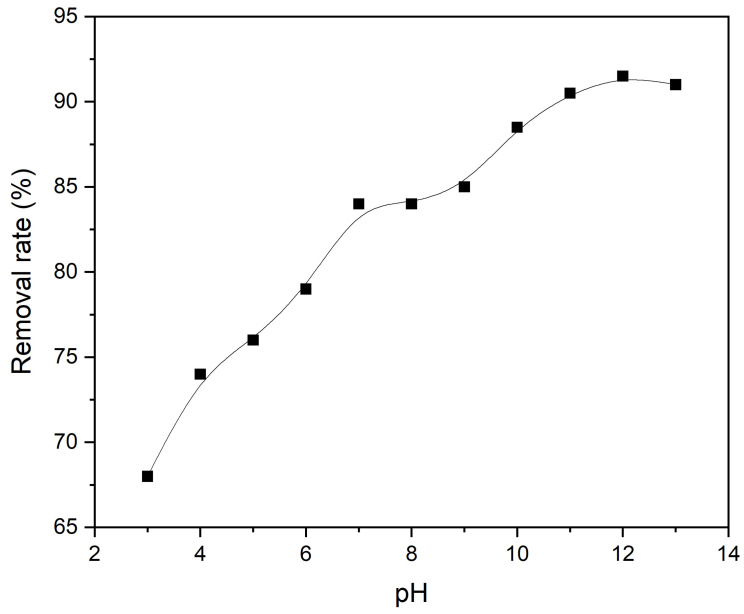
Effect of pH value on adsorption of phenol.

Li et al. found that organically modified montmorillonite’s adsorption of phenol is affected by pH, and thus, the pH of phenol solution should first be studied [32]. The pH of phenolic wastewater is usually in the range of 3 to 13; thus, the phenol removal rate was studied for 30 min on 0.6 g OTMA^+^/hectorite at 303 K and 50 mL with 100 mg/L phenol solution at an initial pH of 3–13 (Figure 8). The results show that the removal rate of phenol is 68.4% at pH 3.0, and it increases with the increase in pH. The removal rate attains the highest 91.6% at pH 12. This result could be attributed to the fact that phenol and OH^−^ reaction, which generates C_6_H_5_O^−^ with the increase in pH and OTMA^+^/hectorite, is superior to adsorbing phenolic ions. In addition, excessively strong acidic or alkaline conditions impair the stability of hectorite, and the material exhibits optimal stability at a pH range of 11 to 12.

**Figure 9:**
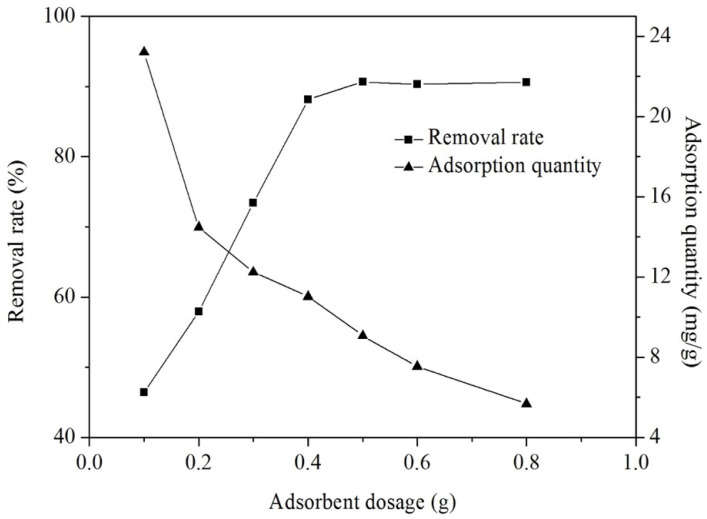
Effect of the adsorbent content on phenol adsorption by OTMA^+^/hectorite.

The adsorption rate of phenol in aqueous solutions was studied with OTMA^+^/hectorite dosages of 0.1, 0.2, 0.3, 0.4, 0.5, 0.6 and 0.8 g at 303 K and 50 mL with 100 mg/L initial phenol solution at pH 12 for 30 min, and the results are shown in Figure 9. The figure shows that the adsorption rate of phenol initially increases and then remains almost unchanged with the increase in the dosage of adsorbent. The phenol removal rate reaches about 92.3% when 0.5 g of adsorbent is used.

### 3.4. Adsorption Isotherm

**Figure 10:**
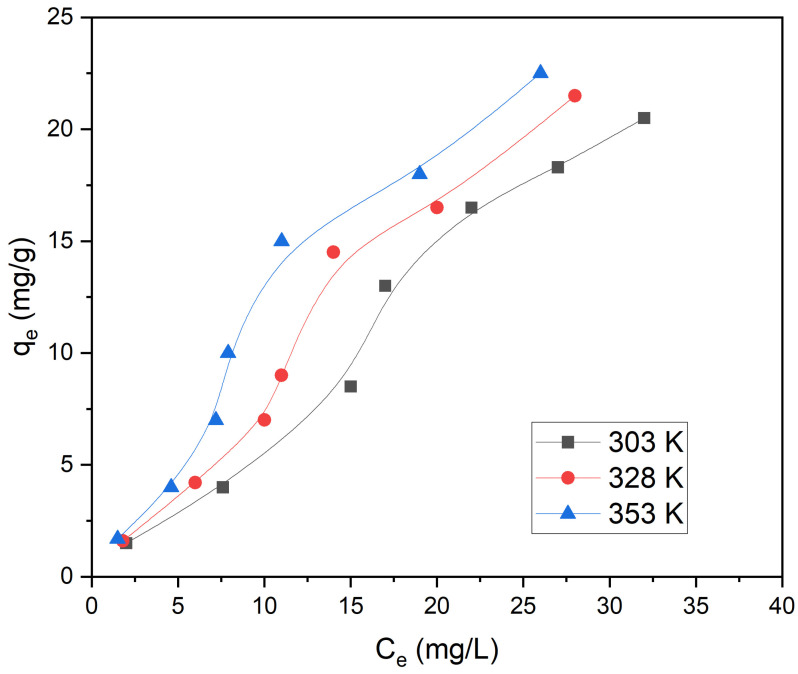
Isotherms of phenol adsorption by OTMA^+^/hectorite.

The adsorption isotherm is key to understanding the equilibrium distribution between adsorbate and the adsorbent at different concentrations. The effect of temperature on phenol adsorption q_e_ and C_e_ on OTMA^+^/hectorite from aqueous solution was studied of 303, 328 and 353 K at pH 12 for 30 min. The adsorption isotherms of phenol from aqueous solution with OTMA^+^/hectorite at the different temperatures are given in Figure 10.

The figures show S-type curves, indicating that there is strong competition between the adsorbing compounds and the solvent molecules for the binding sites [33]. The curves increase steadily, with no obvious plateau, illustrating that OTMA^+^/hectorite presents new surfaces with high attraction for phenol. With the increase in temperature, the adsorption capacities only slightly increase, indicating that temperature has a weak influence on the adsorption of phenol in OTMA^+^/hectorite.

The adsorption data was fitted according to three common isotherm equations of Freundlich, Langmuir and Temkin. The Freundlich isotherm is given in Equation (3), which is fit for non-ideal sorption on heterogeneous surfaces and multilayer sorption. The Langmuir isotherm is expressed by Equation (4), which is fit for monolayer adsorption in a surface containing a finite number of identical sites. The Temkin isotherm is given in Equation (5), which requires that the heat of adsorption of all the adsorbates in layer decreases linearly with coverage due to adsorbent–adsorbate interactions.

(3) $$lnq_{e}=lnK_{F}+\frac{1}{n}lnC_{e}$$

(4) $${\frac{1}{q_{e}}=\frac{1}{q_{m}}+\frac{1}{K_{L}q_{m}C}}_{e}$$

(5) $$q_{e}=BlnA_{T}+BlnC_{e}$$

where C_e_ (mg/L) and q_e_ (mg/g) are the phenol equilibrium concentration and adsorption amount; q_m_ (mg/g) and K_L_ (L/mg) are the maximum adsorption amount and the Langmuir constant. K_F_ (L/g) and n are the Freundlich isotherm parameters related to the adsorption amount and intensity of adsorption. A_T_ is the equilibrium binding constant (L/mg), and B is the Temkin constant associated with the parameter b_T_, which represents the variation in adsorption energy (kJ/mol) according to the following Equation (6).

(6) $$b_{T}=\frac{RT}{B}$$

**Figure 11:**
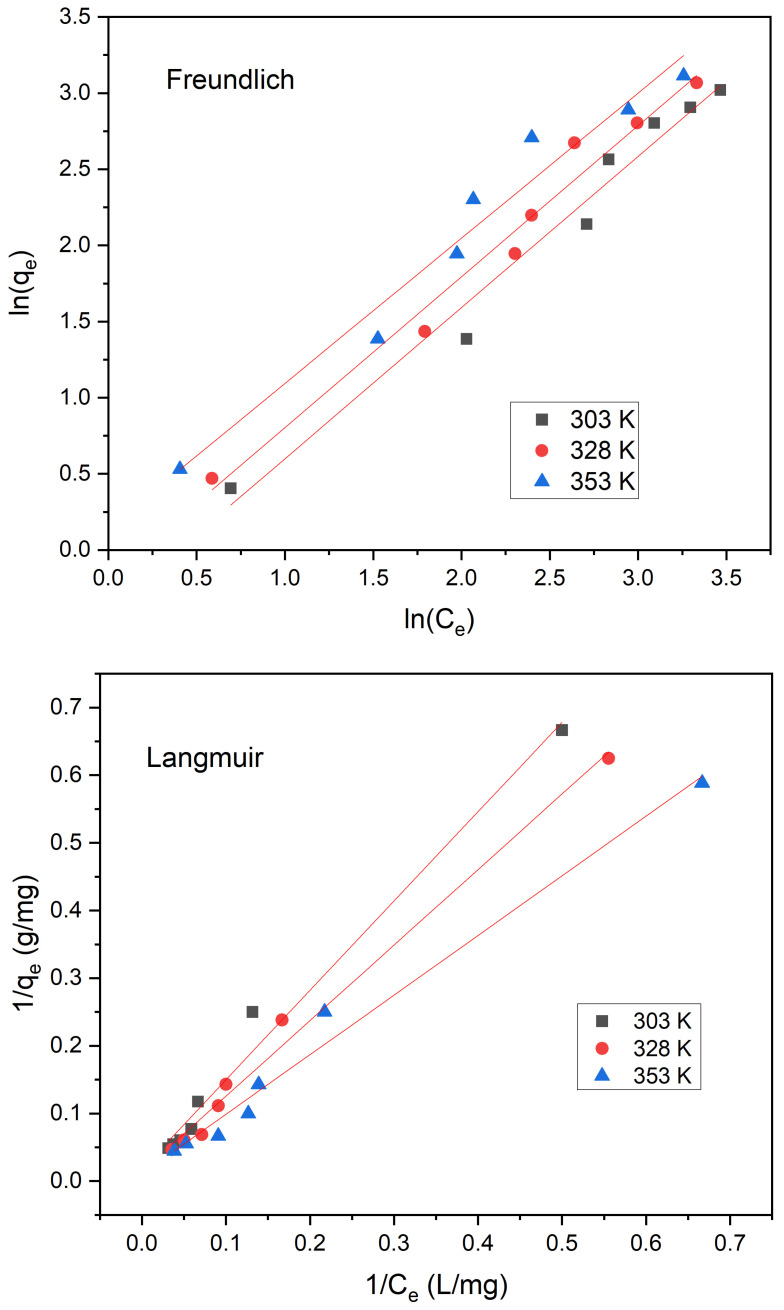

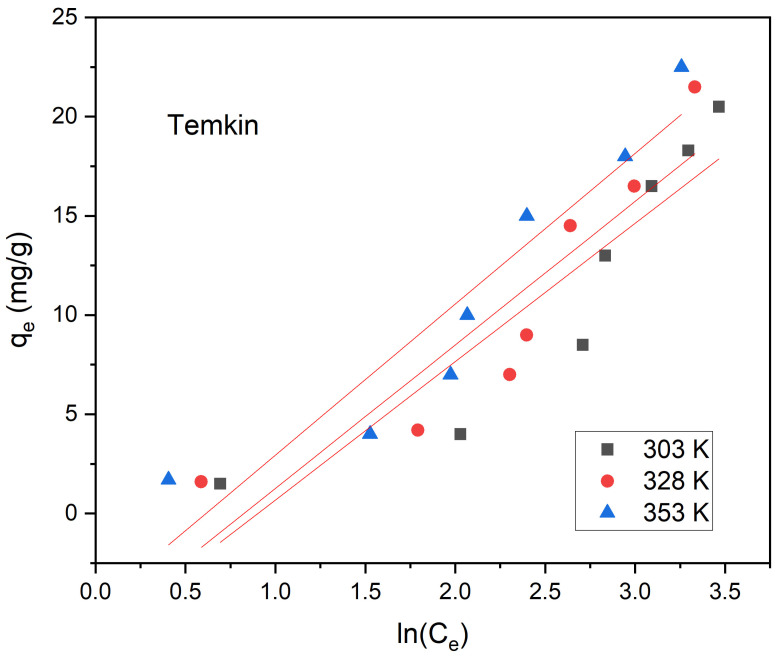
Different isotherm models for adsorption of phenol by OTMA^+^/hectorite.

Based on the adsorption data of phenol at different temperatures, the Freundlich, Langmuir and Temkin isotherm plots are given in Figure 11. The corresponding fitting parameters calculated are shown in Table 2. The appropriate fitting models are in the order Langmuir > Freundlich > Temkin according to R^2^. The results show that the adsorption mechanism is predominantly monolayer adsorption and that the surface of the adsorbent is relatively uniform, with the adsorbate molecules arranged in an orderly manner without multilayer stacking and no interactions between adsorption sites.

As for the Freundlich isotherm, it is reported that values of n less than 1 represent poor adsorptive properties. Therefore, according to the value of n in Table 2, the Freundlich isotherm is also good. These favorable fitting results indicate that the adsorption process is not solely restricted to a monolayer but also involves multilayer adsorption. This differs from studies examining only Freundlich or Temkin isotherms in which the adsorption involved a heterogeneous surface and multilayer adsorption of porous activated carbon, organo-montmorillonite and cellulose-based adsorbent [34,35,36]. The R^2^ of the Temkin isotherm is less than 0.9, which means that the model is not suitable for the adsorption of phenol by OTMA^+^/hectorite [37].

Therefore, the results indicate that in addition to the predominant monolayer adsorption, the phenol adsorption in OTMA^+^/hectorite involves multiple mechanisms, and the S-type isotherm also suggests that cooperative adsorption involves multiple mechanisms [38].

### 3.5. Adsorption Kinetics

**Figure 12:**
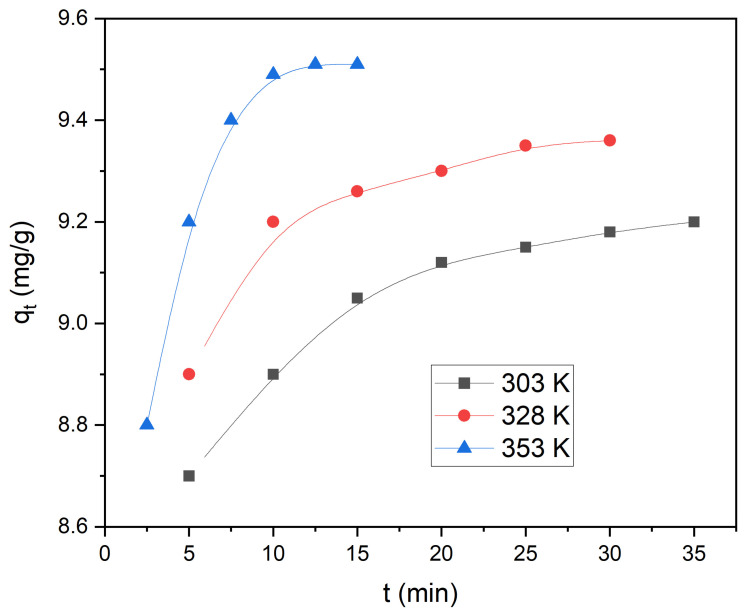
Effect of temperature and time on phenol adsorption by OTMA^+^/hectorite.

Figure 12 shows the effect of temperature and time on the adsorption of phenol by 0.5 g OTMA^+^/hectorite at 50 mL with 100 mg/L initial phenol solution at pH 12 and different temperatures of 303, 328 and 353 K.

The pseudo-first-order and pseudo-second-order kinetic models are generally used to study adsorption, and so they were applied to the experimental data. The pseudo-first-order and pseudo-second-order models are generally expressed as Equations (7) and (8).

q_t_ = q_e_(1 − e^−k_1_t^) (7)

(8) $$q_{t}=\frac{{{k_{2}q}_{e}}^{2}t}{1+k_{2}q_{e}t}$$

where q_t_ (mg/g) is the amount of the phenol adsorbed at t (min); k_1_ (min^−1^) and k_2_ (g/(mg·min)) are the pseudo-first-order and pseudo-second-order rate constants, respectively.

**Figure 13:**
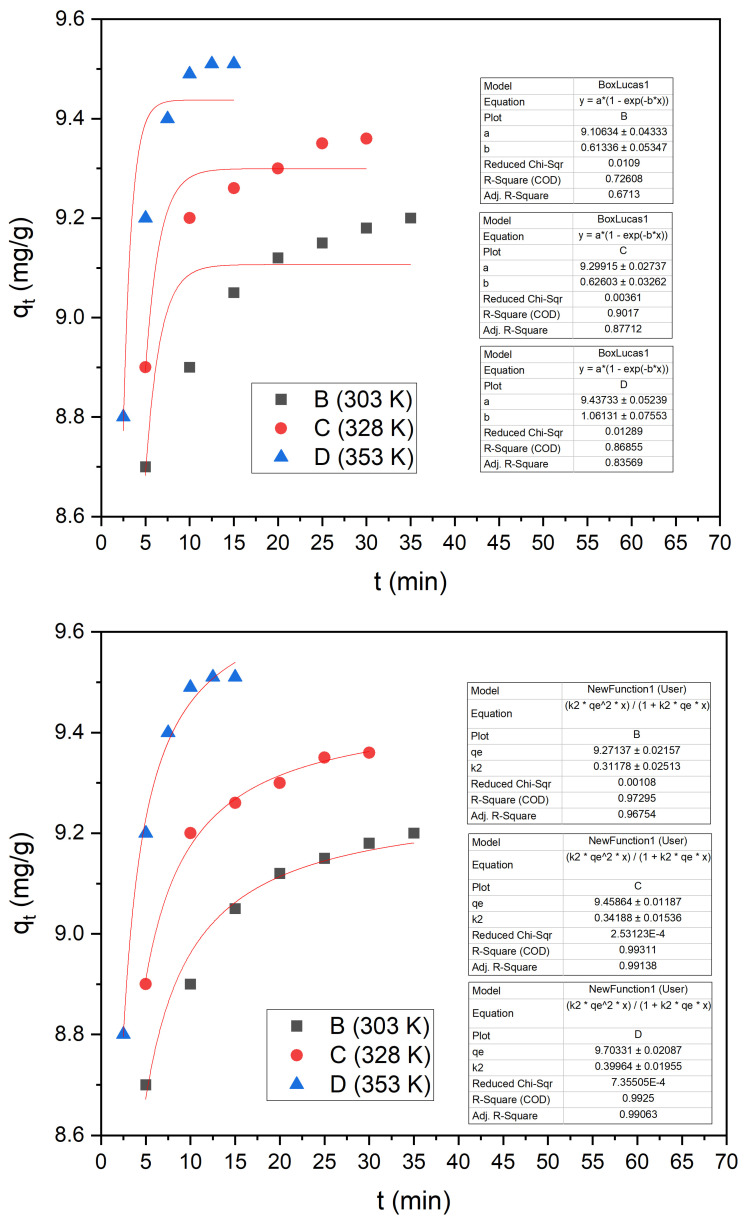
The nonlinear fitting of pseudo-first-order and pseudo-second-order kinetics for the adsorption of phenol by OTMA^+^/hectorite.

The nonlinear fitting was based on the above equations for pseudo-first-order and pseudo-second-order models, as shown in Figure 13. The equation parameters were calculated from the curves, and they are given in Table 3. As for the adsorption, the pseudo-second-order model is better than the pseudo-first-order model because its R^2^ is closer to 1.

The activation energy can be determined with the Arrhenius equation as shown in Equation (9) below with the temperature range from 303 to 353 K.

(9) $$lnk=lnA-\frac{Ea}{RT}$$

**Figure 14:**
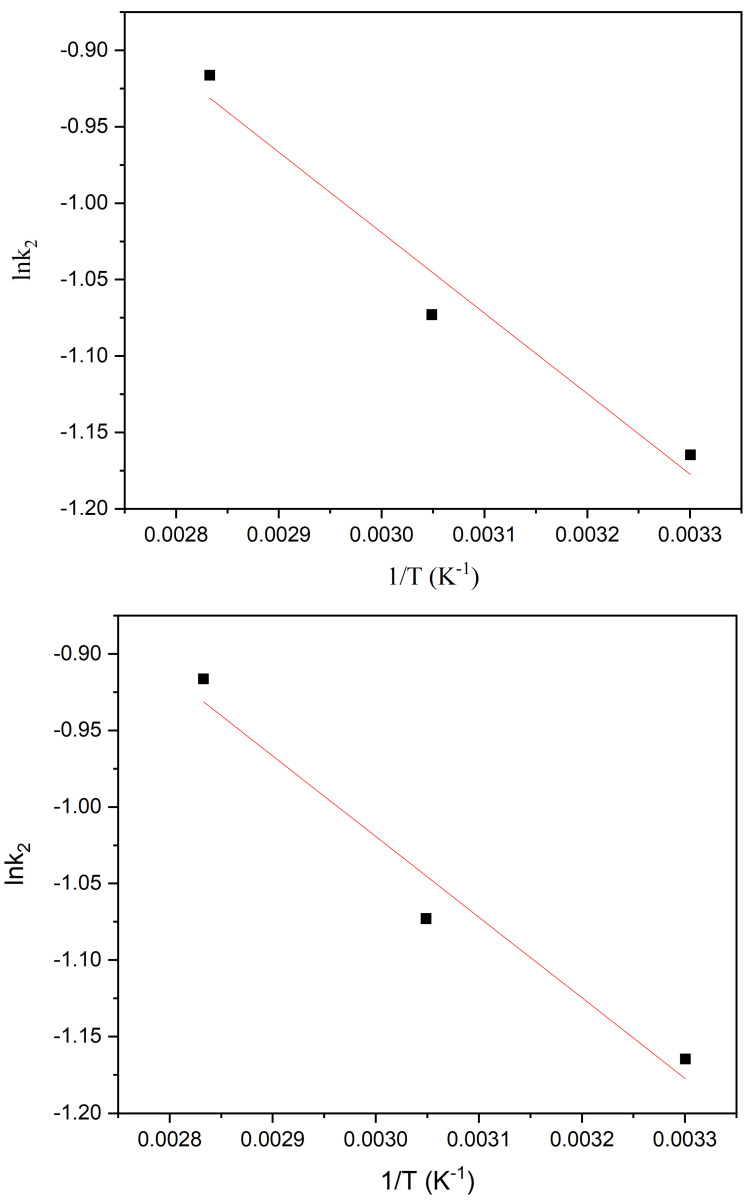
The curve of lnk_2_ vs. 1/T.

The line-fitting relationship between lnk and 1/T shows the apparent reaction activation energy (Ea). Ea, A and R are, respectively, the activation energy (kJ/mol), Arrhenius constant and universal gas constant (8.314 J/(mol·K)). As for the pseudo-second-order model, a plot of lnk_2_ against 1/T gives a straight line, as shown in Figure 14.

According to the intercept of the curve, the activation energy of the adsorption phenol in OTMA^+^/hectorite was 11.15 kJ/mol. The activation energy of physisorption generally ranges from 5 to 40 kJ/mol [39], and therefore, this adsorption process belongs to physisorption. Although the kinetic results are similar to those of activated carbon [40,41,42,43], their mechanisms are different. Activated carbon relies on chemical adsorption, while adsorption on modified hectorite is mainly dominated by physisorption [44].

### 3.6. The Recyclability of OTMA^+^/Hectorite

**Figure 15:**
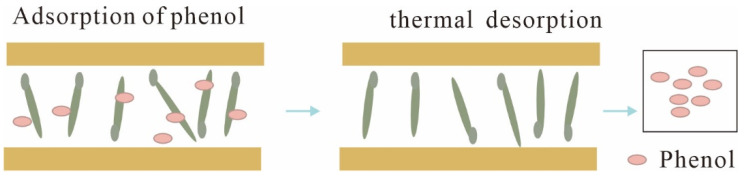
Diagram of adsorbent regeneration process.

Hectorite remains stable at heating temperatures below 450 °C and within a pH range of 6–13. Structural alterations occur during adsorption and desorption processes, which lead to deterioration of its recyclability of adsorption performance. Reutilization is important to industrial application. To determine the stability of the adsorbent, its recycling usage was studied with 0.5 g OTMA^+^/hectorite at 303 K and 50 mL with 100 mg/L initial phenol solution at pH 12. After each usage, the adsorbent was centrifuge-washed and dried in 190 °C for 3 h under vacuum for 3 h to allow the phenol to be volatilized into the gas collection system. Figure 15 presents a schematic diagram of the adsorbent regeneration process.

Table 4 shows the performance of the OTMA^+^/hectorite in four consecutive runs. In the fifth cycle, the removal rate of phenol still reaches 80.1%, demonstrating that the OTMA^+^/hectorite has good stability. After repeated use, the adsorbed substances cannot be completely removed during drying. Repeated usage also causes pore collapse, which reduces the available adsorption space and thus lowers the adsorption capacity.

## 4. Conclusions

Hectorite intercalated with octadecyl trimethylammonium ions was synthesized via one-pot steam synthesis, and the OTMA^+^-modified hectorite has larger layer spacing, surface area and lipophilicity. Therefore, the OTMA^+^-modified hectorite was used as adsorbent to remove phenol from aqueous solution as its capacity is much larger than that of similar clay minerals. In the synthesis process, the organic cations can easily enter the interlayer of hectorite because the organic intercalation and the formation of hectorite occur simultaneously with thorough mixing of the reactants of different mole ratios of lithium, magnesium, silicon and organically modified quaternary ammonium salt. This method simplifies the synthesis process and reduces production costs.

The phenol in aqueous solution was removed through adsorption using the organically modified hectorite, and the isotherms and kinetics were studied. Regarding the adsorption isotherm, the Langmuir and Freundlich models are appropriate, which indicates that in addition to the predominant monolayer adsorption, the phenol adsorption in OTMA^+^/hectorite involves multiple mechanisms. The pseudo-second-order reaction rate model fit the kinetic data, and the activation energy (Ea) was about 11.15 kJ/mol. The OTMA^+^/hectorite could be recycled and reused after regeneration. In this work, the static adsorption process of single-component phenol on modified lithium magnesium silicate was investigated. Given the complex and variable compositions of actual wastewater, future research can focus on the adsorption performance of organically modified lithium magnesium silicate in real wastewater.

## Figures and Tables

**Table 1:** BET surface area, pore diameter values and pore volume.

Adsorbent	S_BET_ (m^2^/g)	Pore Diameter (nm)	Pore Volume (cm^3^/g)
Hectorite	260.36	3.22	0.15
OTMA^+^/hectorite	395.81	4.18	0.44

**Table 2:** The Freundlich, Langmuir and Temkin constants for the adsorption of phenol by OTMA^+^/hectorite.

T (K)	Freundlich			Langmuir		Temkin			
	K_F_ (L/mg)	n	R^2^	K_L_ (L/g)	q_m_ (mg/g)	R^2^	b_T_ (kJ/mol)	A_T_ (L/mg)	R^2^
303	0.645	1.007	0.976	0.0135	55.96	0.985	0.361	0.406	0.838
328	0.829	1.009	0. 976	0.0126	71.225	0.990	0.377	0.438	0.830
353	1.152	1.049	0.963	0.012	94.697	0.984	0.386	0.542	0.887

**Table 3:** The kinetic parameter constants for the adsorption of phenol by OTMA^+^/hectorite.

T (K)	Pseudo-First-Order Model			Pseudo-Second-Order Model		
	q_e_ (mg/g)	k_1_ (min^−1^)	R^2^	q_e_ (mg/g)	k_2_ (g/(mg·min))	R^2^
303	9.106	0.613	0.726	9.271	0.312	0.973
328	9.299	0.626	0.902	9.459	0.342	0.993
353	9.437	1.061	0.868	9.703	0.400	0.992

**Table 4:** The reusability of OTMA^+^/hectorite to the adsorption of phenol.

Cycles	1	2	3	4	5
Removal rate (%)	92.3	91.6	88.5	85.7	80.1

## Data Availability

The original contributions presented in this study are included in the article. Further inquiries can be directed to the corresponding author.
